# Evaluation of efficacy and safety of sequential antibody drug conjugates (ADCs) in human epidermal growth factor 2 (HER2)-negative metastatic breast cancer

**DOI:** 10.1007/s10549-025-07818-z

**Published:** 2025-09-11

**Authors:** Sara Nezirevic, Carey Anders, Susan Dent, Rani Bansal, Lexie Zidanyue Yang, Alaattin Erkanli, Heather Moore

**Affiliations:** 1 Department of Pharmaceutical Services, Vanderbilt-Ingram Cancer Center, 1301 Medical Center Drive, Nashville, TN 37232 USA; 2 Department of Medicine, Wilmot Cancer Institute, University of Rochester, Rochester, NY USA; 3 Duke Cancer Institute, Department of Medicine, Duke University, Durham, NC USA; 4 Department of Biostatistics and Bioinformatics, Duke University Hospital, Durham, NC USA; 5 Department of Pharmacy, Duke University Hospital, Durham, NC USA

**Keywords:** Antibody drug conjugate, Sacituzumab, Trastuzumab, Breast

## Abstract

**Purpose:**

Limited data is available assessing sequencing of antibody drug conjugates (ADCs) in patients with hormone receptor-positive (HR +), human epidermal growth factor 2 (HER2)-negative, HER2-low, and triple-negative metastatic breast cancer (MBC), including patients with brain metastases (BrM) or leptomeningeal disease (LMD). This study assesses the efficacy and safety of sequential sacituzumab govitecan (SG) and trastuzumab deruxtecan (T-DXd) in MBC and impact on chemotherapy (CTX).

**Methods:**

This is a single-center, retrospective, cohort study in adult patients with HR + , HER2-negative, or low MBC who received T-DXd and/or SG.

**Results:**

A total of 112 patients were divided into three cohorts: ADCs given sequentially (cohort A), ADC then CTX (cohort B), or CTX between ADCs (cohort C). The median progression-free survival (mPFS) in cohort A was 4.5 months for SG before T-DXd and 3.1 months for T-DXd before SG. In cohort B, mPFS was 3.1 months for CTX following T-DXd. For CTX following SG, mPFS for CTX was 2.5 months. In patients who received both ADCs, PFS was 2.1 months. In cohort C, mPFS for SG following T-DXd and CTX was 2.1 months and 3.3 months for T-DXd following SG and CTX. The mPFS for ADC1 was longer than ADC2 (5.5 months SG, 3.4 months T-DXd). Those with BrM and/or LMD demonstrated stable disease.

**Conclusion:**

Sequential administration of ADCs results in a shorter PFS. CTX efficacy is impacted by prior ADC administration. Outcomes for patients with BrM and LMD do not differ for those without recurrence to the brain.

**Supplementary Information:**

The online version contains supplementary material available at 10.1007/s10549-025-07818-z.

## Introduction

Of the 290,000 cases of breast cancer that are diagnosed per year in the United States, roughly 6% of patients will present with advanced or metastatic breast cancer [1]. The 5-year survival for metastatic breast cancer in females is 31.0% [1]. For patients with hormone receptor (HR)-positive, human epidermal growth factor receptor 2 (HER2)-negative metastatic disease, treatment commonly consists of endocrine therapy in combination with targeted agents such as cyclin-dependent kinase (CDK) 4/6, PI3K, or AKT inhibitors. Few targeted agents are available for patients with hormone receptor-negative, HER2-negative (triple-negative) metastatic disease. Recent studies evaluating antibody drug conjugates (ADCs), including sacituzumab govitecan (SG), and trastuzumab deruxtecan (T-DXd) have demonstrated significantly improved progression-free survival (PFS) and overall survival (OS) in advanced breast cancer. The TROPiCS02 trial investigated the use of SG in patients with endocrine-resistant HR +/HER2-negative locally inoperable or metastatic breast cancer. The study demonstrated a median overall survival (OS) of 14.5 months in the SG arm versus 11.2 months in the physician’s choice arm, with a median progression-free survival (PFS) of 5.5 months versus 4.0 months [2]. SG previously received FDA approval in the triple-negative metastatic disease setting based on increased PFS and OS reported at 5.6 months and 12.1 months, respectively, from the ASCENT trial, and 5.5 months and 13.0 months, respectively, in the IMMU-132 trial [3, 4]. Ultimately, SG was approved for patients with unresectable locally advanced or metastatic HR-positive, HER2-negative breast cancer who received endocrine-based therapy and at least two additional chemotherapies in the metastatic setting, expanding its indication and use.

Destiny-Breast04 evaluated the use of T-DXd in in patients with HER2-low (IHC 1 + or 2 +) metastatic breast cancer and demonstrated that T-DXd significantly improved PFS and OS. T-DXd improved PFS by 4.7 months and median OS by 6.6 months in the HR + cohort and by 5.6 months and 9.9 months, respectively, in the HR- cohort. Additionally, the risk of disease progression or death was decreased with T-DXd by 50% with a hazard ratio of 0.50 [5]. As a result, T-DXd was approved for patients with unresectable or metastatic HER2-low breast cancer who have received one prior line of chemotherapy in the metastatic setting or developed disease recurrence during or within six months of completing adjuvant chemotherapy.

ADCs have drastically changed the treatment paradigm of breast cancer in the metastatic setting. Antibody–drug conjugates (ADCs) consist of a highly selective monoclonal antibody (mAb) with a cytotoxic payload that stimulates target cell death via a linker that is stable in circulation [6]. ADCs can efficiently target antigen-positive (Ag +) cells, however antigen-negative (Ag−) cells remain unexposed to the cytotoxic payload. The chemistry of the linker and cytotoxic drug determines how and when the payload is released, and if the drug can diffuse into surrounding Ag- cells. This diffusion is known as the bystander effect, which allows the early release of cytotoxins from the linker with the opportunity to reach cells surrounding the tumor cells [7]. Both SG and T-DXd have demonstrated this bystander effect. SG is composed of humanized RS7 (hRS7) anti-Trop-2 monoclonal antibody that delivers SN-38, the active metabolite of irinotecan and a topoisomerase inhibitor, to Trop-2-expressing cells which is expressed on 93% of triple-negative and 50% of HR + breast cancer cells [8, 9]. T-DXd utilizes a humanized anti-HER2 monoclonal antibody, trastuzumab, linked to deruxtecan, a derivative of exatecan (camptothecin structural analog) and topoisomerase I inhibitor payload to target HER2 cells [10]. Despite the profound efficacy of ADCs, there is a concern for resistance or compromised efficacy when sequencing therapies that may utilize cytotoxic payloads with similar mechanisms of action.

The TROPION-PanTumor01 study evaluated the use of datopotamab deruxtecan, an ADC directed at Trop-2 linked to deruxtecan for the treatment of relapsed and refractory advanced triple-negative breast cancer (TNBC). An overall response rate (ORR) of 31.8% was observed in all patients, and 40% in patients who were treatment-naïve to topoisomerase I inhibitor-based ADC therapies. Of the patients in the study, 31.8% of patients had received prior treatment with a topoisomerase 1 inhibitor-based antibody. Datopotamab-DXd demonstrated a confirmed ORR 26.8% in the HR +/HER2-negative cohort and 31.8% in those with TNBC. The responses were durable with a duration of response (DOR) not evaluable in the HR +/HER2-negative group and 16.8 months in the TNBC group. Overall, in those who were treatment-naïve, a higher ORR and longer median OS and PFS were reported suggesting that tumors may start to develop resistance to topoisomerase I inhibition through previous exposure. Additionally, HR +/HER2-negative patients had 2 (range 1–6) previous lines of chemotherapy and more than a 30% decrease in measurable disease [11, 12]. This may suggest that with the cumulative effect of previous cytotoxic agents, there may be a level of increased resistance to topoisomerase I inhibition.

Given the availability of multiple ADCs, the sequencing of these drugs has been questioned and poses a concern of potential resistance when given sequentially. This retrospective study aims to assess the efficacy, safety, and tolerability of sequential ADCs with similar cytotoxic mechanisms (SG and T-DXd) in the HER2-negative metastatic breast cancer setting and determine if the efficacy of sequential conventional cytotoxic chemotherapy may be impacted by ADC use.

## Methods

### Design and setting

This single-center, retrospective, cohort study assessed the efficacy, safety, and tolerability of sequential ADCs in the HER2-negative metastatic breast cancer setting. Adult patients ≥ 18 years of age with a diagnosis of HR +, HER2-low, or triple-negative advanced/metastatic breast cancer who received T-DXd and/or SG between April 2021 and October 2023 at Duke Cancer Center were included. Patients were excluded if they had not received prescribed therapy from Duke Cancer Center and or their clinical care was managed locally. Patients were divided into three cohorts, depending on if patients had received ADCs sequentially, ADC then chemotherapy thereafter, or if they received chemotherapy in between two ADCs.

### Data source and data collection

Data was collected for all patients through a retrospective chart review and managed using the Research Electronic Data Capture (REDCap) hosted at Duke University. REDCap is a secure, web-based software platform designed to support data capture for research studies. HER2 status was assessed using reported IHC from Duke pathology. Routine clinical and laboratory assessments, including safety endpoints and dose modifications, were conducted with every infusion.

### Measures

The efficacy endpoints include progression-free survival (PFS) and intracranial and extracranial response rate, with PFS calculated as the time in months from therapy initiation to progression or death from any cause. The primary endpoints included PFS of SG following T-DXd and PFS of T-DXd following SG. Secondary endpoints included PFS of SG and T-DXd initial ADC therapy, PFS of first non-ADC therapy following T-DXd OR SG, PFS of first non-ADC therapy following receipt of both T-DXd AND SG, and intracranial and extracranial response rate after starting ADC in patients with brain metastases and/or leptomeningeal disease (LMD) after sequential ADC therapy. The safety endpoint was the real-world adverse event profile of ADC (SG or T-DXd). The intracranial and extracranial response rate was measured by Response Evaluation Criteria in Solid Tumors (RECIST) criteria defined as complete response (CR), partial response (PR), or stable disease (SD). The adverse events were graded using the Common Terminology Criteria for Adverse Events (CTCAE) version 5.0.

### Statistical analysis

Kaplan–Meier curves and estimates were used to describe the time until progression/death event and the event-free survival rates. Patients with no events were censored at the end of data collection, December 1, 2023. This analysis of PFS was performed for all patients and stratified by treatment patterns. Descriptive statistics were utilized to summarize the demographics, clinical characteristics, and outcomes of the patients who received ADCs as outlined above. Continuous variables were summarized with means, medians, standard deviations, Q1, Q3, and ranges. Categorical variables were summarized with frequency (counts) and percentages. All statistical analyses were performed in SAS version 9.4 (SAS institute Inc., Cary, NC).

## Results

### Patient characteristics

**Fig. 1:**
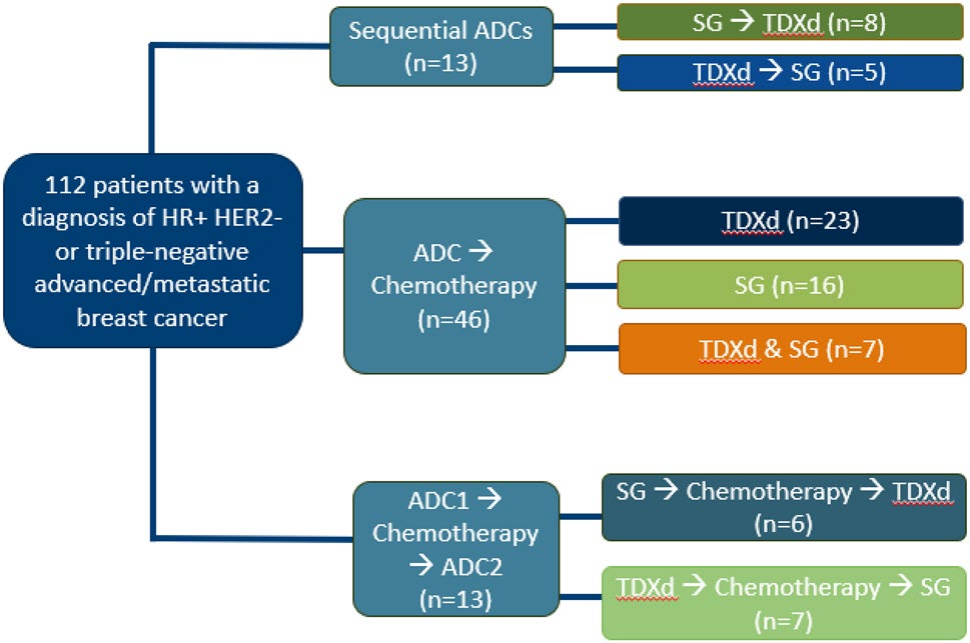
Patient cohorts

A total of 123 patients with HR +/HER2-negative or triple-negative breast cancer were screened for potential inclusion in the study from April 1, 2021, to October 31, 2023, (Figure S1). Exclusion criteria consisted of 11 patients who were treated at an outside facility. Of the 112 patients included who had received T-DXd and/or SG, 59 (52.6%) patients had HR + MBC, and 43 (38.3) with TNBC (Table 1). Median age was 61.5 years old (IQR 52–68), with 55.4% of patients of Caucasian race and 35.7% of African American race. Patients were predominantly post-menopausal (92.9%). Most patients had an Eastern Cooperative Oncology Group (ECOG) performance status less than or equal to 2 (93.8%), with the majority having an ECOG performance score of 0 − 1 (75.9%). Most patients had two or more sites of metastatic disease (96.4%), with the most common sites being bone (67%), lung (59.8%), and liver (58%). Approximately 22.3% and 2.7% of patients had brain and/or LMD metastases, respectively. Patients had a median of 2 lines of therapy for metastatic disease prior to the initiation of an ADC (IQR 1–3), with triple-negative patients commonly receiving capecitabine (51.8%), paclitaxel or nab-paclitaxel (29.5%) +/− pembrolizumab if PD-L1 positive (5.4%), and carboplatin with gemcitabine therapy (17.9%) +/− pembrolizumab if PD-L1 positive (11.6%). Patients with HR + metastatic disease had a median of 1 prior line of endocrine therapy (ET) for metastatic disease (IQR 0 − 2), with cyclin-dependent kinase 4/6 (CDK4/6) inhibitors in combination with ET (44.6%) being the most common therapy (Tables S1 and S2). Patients were divided into three cohorts as follows, ADCs given sequentially (*n* = 13), ADC then chemotherapy there after (*n* = 46), or if they received chemotherapy in between two ADCs (*n* = 13) (Fig. 1).

**Table 1:** Patient demographics and characteristics

Patient demographics	Total (*n* = 112)
Age, median (IQR)	61.5 (52–68)
*Gender—no. (%)*	
Female	110 (98.2)
Male	2 (1.7)
*Race—no. (%)*	
Caucasian	62 (55.4)
African American	40 (35.7)
American Indian or Alaska Native	3 (2.7)
Asian	2 (1.8)
Other	4 (3.6)
*Menopausal status—no. (%)*	
Pre-menopausal	5 (4.5)
Post-menopausal	104 (92.9)
N/A	3 (2.7)
*ECOG—no. (%)*	
0	33 (29.5)
1	52 (46.4)
2	20 (17.9)
3	5 (4.5)
Unknown	2 (1.8)
*Histology—no. (%)*	
Ductal	83 (74.1)
Lobular	12 (10.7)
Mixed	12 (10.7)
Unknown	5 (4.5)
*Grade—no. (%)*	
1	3 (2.7)
2	37 (33.0)
3	63 (56.3)
Unknown	9 (8.0)
Hormone receptor positive—no. (%)	59 (52.6)
Hormone receptor negative—no. (%)	53 (47.3)
HER2-low—no. (%)	32 (28.5)
HER2 0—no. (%)	16 (14.3)
HER2 1 +—no. (%)	64 (57.1)
*Metastases—no. (%)*	
Bone	75 (67.0)
Lung	67 (59.8)
Liver	65 (58.0)
Brain	25 (22.3)
Lymphangitic carcinomatosis	20 (17.9)
Skin	12 (10.7)
Locally advanced	9 (8.0)
Leptomeningeal disease	3 (2.6)
Other	71 (63.4)
*Number of metastatic sites—no. (%)*	
1	4 (3.6)
2	26 (23.2)
3	44 (39.3)
4 +	38 (33.9)
Total lines of therapy (prior to ADC), median (IQR)—no	2.0 (1.0, 3.0)
Total lines of endocrine therapy, median (IQR)—no	1.0 (0.0, 2.0)

### Efficacy

**Fig. 2:**
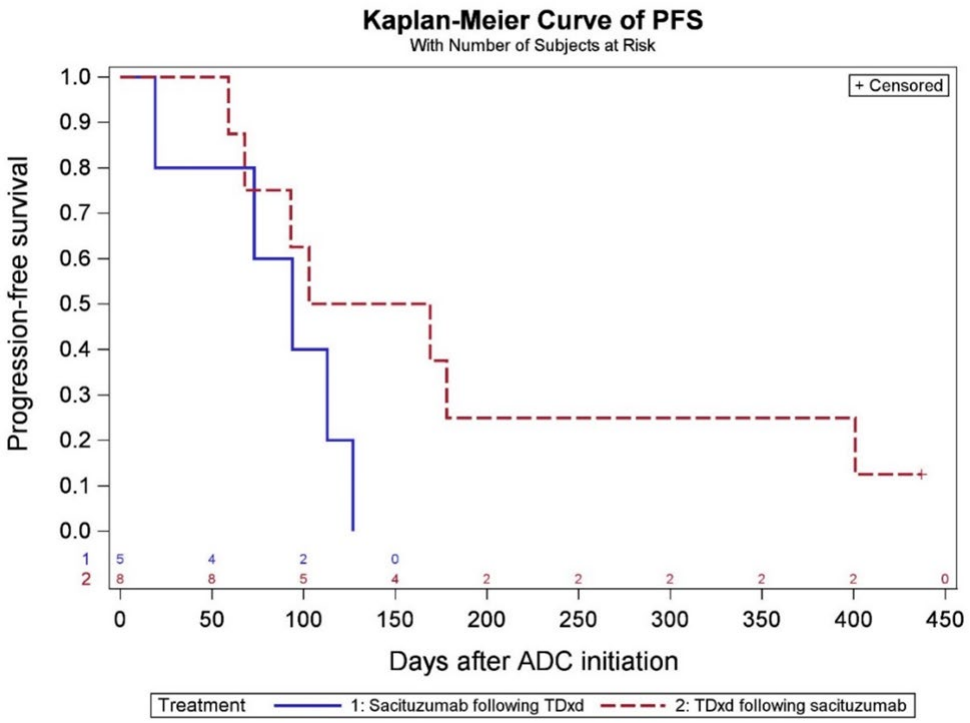
Progression-free survival of sequential ADCs

**Fig. 3:**
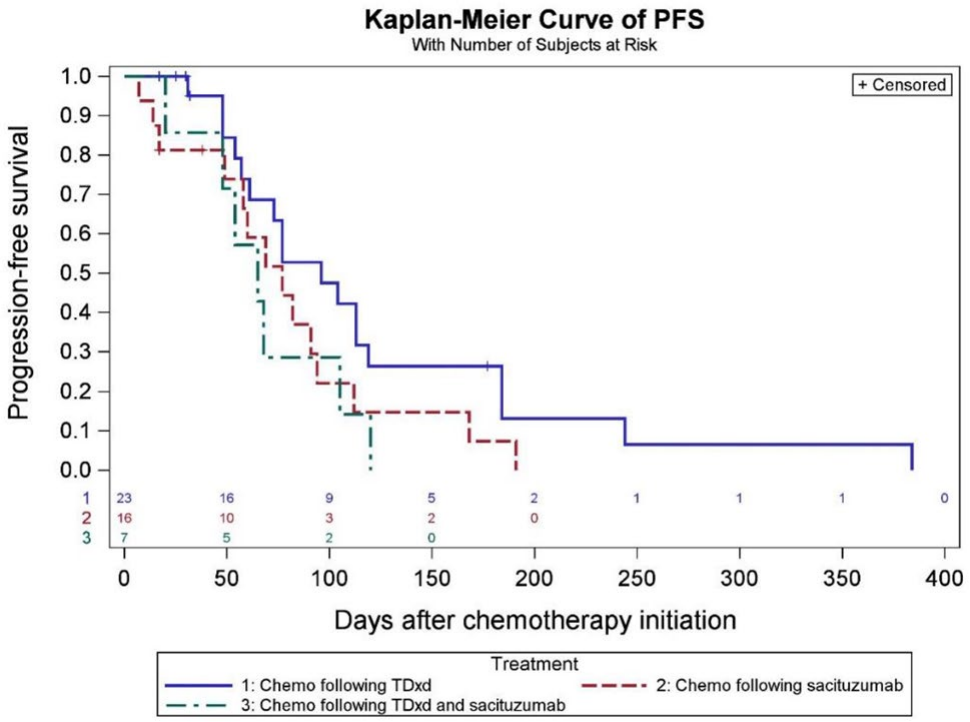
Progression-free survival of chemotherapy following ADC

**Fig. 4:**
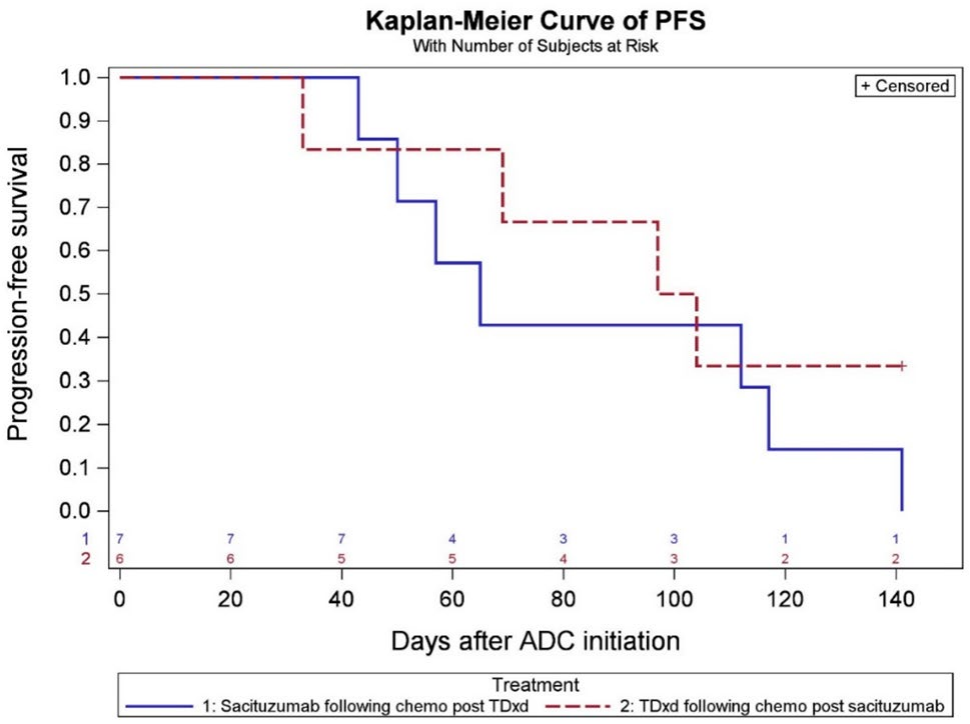
Progression-free survival of ADC2 following chemotherapy and ADC1

**Fig. 5:**
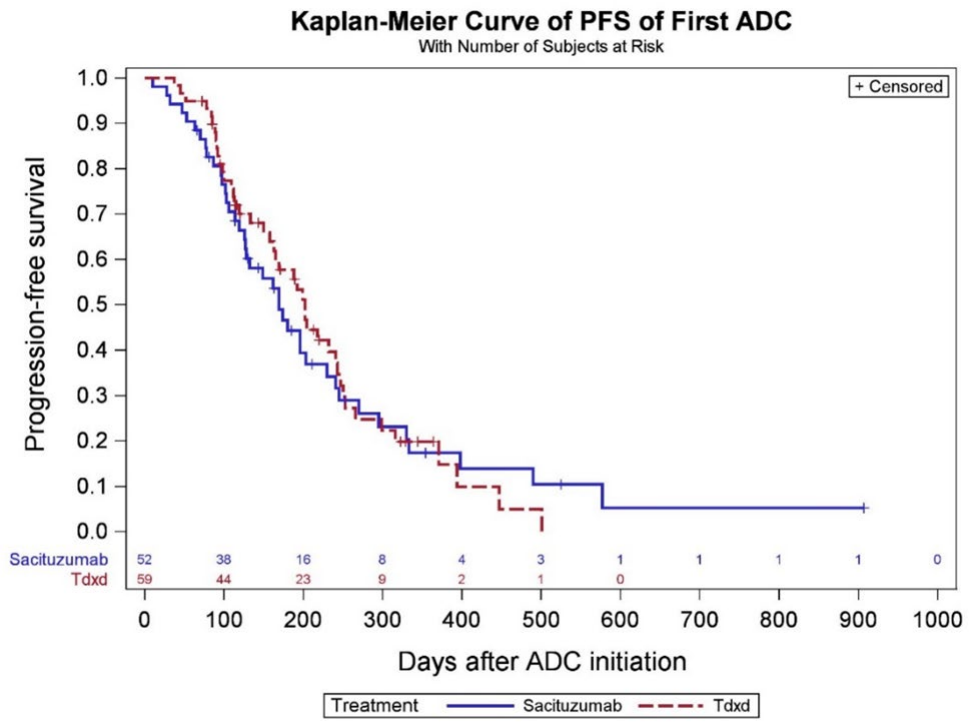
Progression-free survival of first ADC

**Fig. 6:**
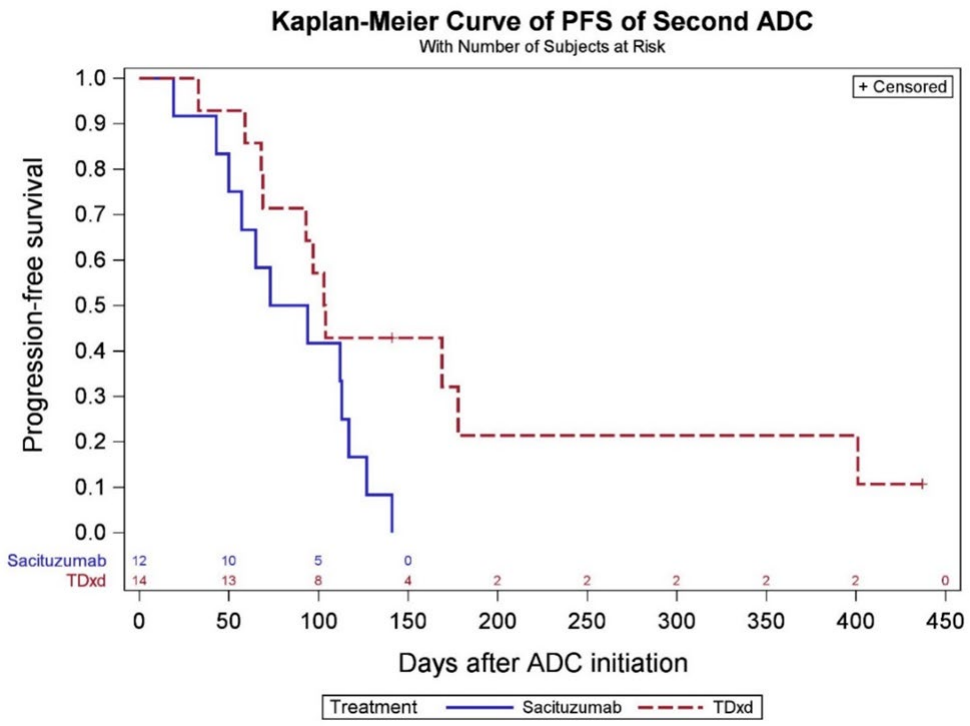
Progression-free survival of second ADC

At the data cutoff date (December 1 st, 2023), the median PFS in the sequential ADCs cohort was 4.5 months in the SG prior to T-DXd group and 3.1 months for T-DXd prior to SG group (Fig. 2). In the chemotherapy following T-DXd group, the median PFS was 3.1 months. Among patients who received chemotherapy following SG, the median PFS was 2.5 months, whereas in patients who received both ADCs, PFS was 2.1 months with subsequent chemotherapy (Fig. 3). Among 23 patients who received T-DXd prior, the most common chemotherapy agents administered after ADC1 were liposomal doxorubicin in 10 patients (43.5%), and eribulin in 4 patients (17.4%). A total of 3 patients (18.8%) received eribulin and 6 patients (37.5%) received liposomal doxorubicin after SG. In 7 patients who received both T-DXd and SG sequentially, the most common chemotherapy administered after was capecitabine (28.6%) and carboplatin with gemcitabine (28.6%) (Tables S5 and S6). In 13 patients who received chemotherapy between two ADCs, the median PFS was 2.1 months for SG following T-DXd and chemotherapy, and 3.3 months for T-DXd following SG and chemotherapy, respectively (Fig. 4). The median PFS for ADC1 was longer than ADC2 regardless of sequence order. The median PFS was 6.6 months when T-DXd was ADC1 and 3.4 months when given as ADC2. The median PFS was 5.5 months when SG was ADC1 and 2.7 months when given as ADC2 (Figs. 5 and 6).

There were 12 patients in the T-DXd group and 17 patients in the SG group who had brain metastases. Within the T-DXd group, 4 patients (33.3%) had extracranial response, and 8 patients (66.7%) had intracranial response. In the SG group, 9 patients (52.9%) had extracranial response, and 8 patients (47.1%) had intracranial response. One patient in the T-DXd group and 2 patients in the SG group had LMD. All patients in both groups had stable LMD after ADC administration. In both groups, those with brain metastases and/or LMD all had stable disease with extracranial response after the administration of an ADC (Tables 2 and 3).

**Table 2:** Response rate by brain metastases and LMD in patients who received T-DXd

CNS involvement	Partial response	Stable disease	Progressive disease	Total (*n* = 112 patients)
Extracranial response in brain metastases, *n* (%)	0 (0)	4 (100)	0 (0)	4 (3.5)
Intracranial response in brain metastases, *n* (%)	0 (0)	8 (100)	0 (0)	8 (7.1)
Extracranial response in LMD, *n* (%)	0 (0)	1 (100)	0 (0)	1 (0.8)

**Table 3:** Response rate by brain metastases and LMD in patients who received SG

CNS involvement	Partial response	Stable disease	Progressive disease	Total (*n* = 112 patients)
Extracranial response in brain metastases, *n* (%)	0 (0)	9 (100)	0 (0)	9 (8.0)
Intracranial response in brain metastases, *n* (%)	0 (0)	8 (100)	0 (0)	8 (7.1)
Extracranial response in LMD, *n* (%)	0 (0)	2 (100)	0 (0)	2 (1.7)

### Safety

The median duration of treatment was 4.8 months for patients who received T-DXd and 3.2 months for SG regardless of drug sequencing. A total of 81.3% of patients in the SG group and 64.9% of those in the T-DXd group had at least one adverse event (AE) that emerged or repeated after initiation of SG (Tables 4 and 5). The incidence of a grade 3 or higher AE was 20.8% for T-DXd and 61.5% for SG. The incidence of AEs associated with discontinuation of treatment was 10.7% in the T-DXd group and 2.1% in the SG group while the incidence of AEs associated with dose reductions was 76.8% and 79.2%, respectively.

**Table 4:** Adverse events in T-DXd group

Adverse events	Total (*n* = 112)
Adverse event occurrence—*n*/total patients received T-DXd (%)	48/74 (64.9)
*Neutropenia—n (%)*	12 (10.7)
Grade ≤ 2	9 (75.0)
Grade 3	2 (16.7)
Grade 4	1 (8.3)
*Nausea—n (%)*	36 (32.1)
Grade ≤ 2	33 (91.6)
Grade 3	3 (8.3)
*Vomiting—n (%)*	20 (17.9)
Grade ≤ 2	16 (80.0)
Grade 3	4 (20.0)
*Hypersensitivity reaction—n (%)*	3 (2.7)
Grade 2	3 (2.7)
Interstitial lung disease—*n* (%)	8 (7.1)
Grade ≤ 2	8 (100.0)
Febrile Neutropenia—*n* (%)	–
Thrombocytopenia—*n* (%)	4 (3.6)
Grade ≤ 2	4 (100.0)
Left Ventricular Dysfunction—*n* (%)	–

**Table 5:** Adverse events in SG group

Adverse events	Total (*n* = 112)
Adverse event occurrence—*n*/total patients received SG (%)	52/64 (81.3)
*Neutropenia—n (%)*	32 (28.6)
Grade ≤ 2	14 (43.7)
Grade 3	13 (40.6)
Grade 4	5 (15.6)
*Nausea—n (%)*	32 (28.6)
Grade ≤ 2	31 (91.1)
Grade 3	1 (3.1)
*Vomiting—n (%)*	16 (14.3)
Grade ≤ 2	14 (87.5)
Grade 3	2 (12.5)
*Diarrhea—n (%)*	29 (25.9)
Grade ≤ 2	26 (89.6)
Grade 3	3 (10.3)
*Hypersensitivity reaction—n (%)*	5 (4.5)
Grade ≤ 2	3 (60.0)
Grade 3	2 (40.0)
*Febrile neutropenia—n (%)*	6 (5.4)
Grade 3	6 (100.0)

In the T-DXd group, the most common drug-related AEs of any grade included nausea (32.1%), vomiting (17.9%), and neutropenia (10.7%), all of which were more frequent than in the SG group except for vomiting. Most common AEs of any grade for those who received SG were nausea (28.6%), neutropenia (28.6%), vomiting, (14.3%), and diarrhea (25.9%). In the T-DXd group, the most common AEs of grade 3 or higher were vomiting (20.0%), neutropenia (25.0%), and nausea (8.3%). The most common AEs of grade 3 or higher in the SG group were febrile neutropenia (100.0%), neutropenia (56.3%), and diarrhea (10.3%). Repeated adverse events occurred in 4 patients (8.3%) who received T-DXd and 7 patients (13.5%) in the SG group. Repeated AEs were nausea (4.2%), neutropenia (2.1%), and vomiting (2.1%) in the T-DXd group, and neutropenia (5.8%), diarrhea (5.8%), and nausea (1.9%) in the SG group. Of the repeated AEs, one grade 3 event (100.0%) of neutropenia occurred in the T-DXd group and two grade 3 events (66.6%) of neutropenia occurred in the SG group (Tables S3 and S4).

Drug-related interstitial lung disease (ILD) occurred in 8 patients (7.1%) who received T-DXd, including 4 with a grade 1 event (50.0%), and 4 patients with a grade 2 event (50.0%). No grade 3 or higher adverse events were reported for ILD. No left ventricular dysfunction was reported in patients who received T-DXd.

## Discussion

This study represents an analysis of sequential use of ADCs, T-DXd, and SG, in patients with HER2-negative, and HER2-low metastatic breast cancer in a real-world practice setting. The sequential administration of SG following T-DXd resulted in a lower median PFS in comparison to SG given prior to T-DXd. The median PFS was longer when patients received chemotherapy after only 1 ADC agent when compared to receiving both T-DXd and SG. The PFS was longer for ADC1 than ADC2 regardless of sequence order. Patients treated with T-DXd and SG demonstrated a comparable PFS to that reported in the existing literature. The safety profile of T-DXd and SG in each cohort had low incidences of grade ≥ 3 adverse events, dose modifications, and treatment discontinuations. No treatment related deaths were reported. While nausea was common, it was generally manageable. Hematologic adverse events were reported, and commonly grade ≥ 3 adverse events in both T-DXd and SG groups. No differences in toxicities were observed among differing sequence order of ADCs.

The use of ADCs has continued to rise because of significant improvement in PFS and OS in patients with HER2-negative and HER2-low metastatic breast cancer. However, with increased use, the optimal sequencing of these agents continues to be a challenge. As seen in TROPION-PanTumor01, among patients with triple-negative metastatic breast cancer who had received ADCs sequentially, the median PFS on the first ADC (ADC1) was significantly longer at 7.55 months compared to 2.53 months with the second ADC (ADC2). Cross-resistance was also present in 53.1% of cases [11]. Mechanisms of ADC resistance can be categorized as a change in antigen expression, change in ADC processing and resistance, and efflux of the ADC payload [13, 14]. However, available data is limited for assessing the optimal sequencing of ADCs and the impact their use may have on the efficacy of therapy. Additionally, in TROPION-PanTumor01, there was a higher ORR and longer median OS and PFS in patients with TNBC who were naïve to topoisomerase I inhibitors. This was compared to the overall TNBC population, which suggests a level of resistance that may develop to topoisomerase I inhibition through previous exposure [12].

In our study, patients who received ADC1 with no prior exposure to an ADC had a longer median PFS compared to patients who received ADC2 after ADC1. This was regardless of which ADC was administered first. Given FDA approval of datopotamab-DXd in HR +, HER2-negative disease, understanding optimal sequencing among ADCs and biomarkers to guide treatment sequencing will be imperative.

As a result of Destiny Breast-04, T-DXd is approved for patients with unresectable or metastatic HER2-low breast cancer who have received one prior line of chemotherapy in the metastatic setting or developed disease recurrence during or within six months of completing adjuvant chemotherapy. Additionally, as Destiny Breast-06, assessing the benefit of T-DXd in HER2 “ultra-low” has shown benefit, the anticipated use of T-DXd in a larger patient population is likely, contributing to the importance of ADC sequencing. As a result of TROPiCs-02 data, SG is approved for patients with unresectable locally advanced or metastatic HR-positive, HER2-negative breast cancer who received endocrine-based therapy and at least two additional chemotherapies in the metastatic setting. With these approved indications, T-DXd is commonly administered prior to SG in patients with HR +, HER2-low metastatic breast cancer. The PFS within our trial was longer when patients received SG prior to T-DXd (4.5 months) sequentially versus T-DXd followed by SG (3.1 months). This was in our TNBC cohort and may continue to reinforce the use of SG prior to T-DXd in the triple-negative setting which has remained controversial despite a larger patient population and more mature data with the ASCENT trial compared to the small cohort of triple-negative patients and only subgroup analyses from Destiny-Breast04.

Data in the literature regarding ADC sequencing is limited. Our data is consistent with real-world overall survival (rwOS) which has previously been reported by Mahtani et al. with similar results in patients with HER2-low metastatic breast cancer receiving T-DXd and SG sequentially. The rwOS was reported to be longer for ADC1 in those who received SG prior to T-DXd (78.3% versus 42.8%) in comparison to those who received T-DXd prior to SG (54.5% versus 21.1%) [15]. Morganti et al. analyzed patients with HER2-negative MBC who received ≥ 2 ADCs and had ≥ 6 months follow up and evaluated time to next treatment (TTNT). Similar TTNT was observed with ADC1 and ADC2. The median TTNT for ADC1 was reported at 4.34 months in the overall population, 4.66 months in HR + patients, and 4.11 months in the TNBC cohort. For ADC2, the median TTNT was 5.39 months in the overall population, 5.62 months for HR +, and 5.29 months in the TNBC cohort [16]. Huppert et al. reported the time to failure (TTF) of SG (ADC1) to be longer than T-DXd (ADC2) when given sequentially (6.3 months vs 3.6 months). Real-world response rate was reported at 77.3% for SG (ADC1) and 8/23 (34.8%) for T-DXd (ADC2). For T-DXd (ADC1) median TTF was 5.3 months and 2.1 months for SG (ADC2), with a real-world response rate of 46.9% for T-DXd (ADC1) and 17.2% for SG (ADC2) [17]. Ongoing research is needed to continue to assess biomarkers in guiding optimal sequencing of these agents.

As the risk of brain metastases is high within TNBC and there is limited representation within clinical trials, it is important to understand the efficacy and impact of ADCs in this population. In TROPiCs-02, a similar clinical benefit in PFS and OS with the use of SG in patients with metastatic TNBC was observed when comparing patients with or without brain metastases [2]. While in HER2 + breast cancer patients with brain metastases, the TUXEDO trial, assessed the use of T-DXd for intracranial efficacy. Two patients (13.3%) had a complete intracranial response, nine (60%) had a partial intracranial response and three (20%) had stable disease as the best intracranial response, with a best overall intracranial response rate of 73.3%, confirming T-DXd’s ability to penetrate the CNS [18]. T-DXd showed substantial extracranial activity in HER2 + MBC patients who were heavily pretreated. An ORR of 63.9% was reported in the DESTINY-Breast03 trial after the administrated of T-DXd [19]. Activity in patients with stable brain metastases was observed with T-DXd and led to a PFS of 87.5%, and a 46.2% ORR in the DEBRAH trial [20]. The response rate in patients with brain metastases and/or LMD did not seem to differ to those without CNS involvement in our trial. All patients who received an ADC within our trial had stable response and intracranial and/or extracranial response. These results further support the clinically relevant intracranial and extracranial activity of SG and T-DXd in patients with HR + and TNBC breast cancer.

Limitations exist within this study given it is a single-center, retrospective design with a small sample size, thus limiting the concrete conclusions that can be drawn from this data. A large sample size could have permitted a reasonable unadjusted comparison of PFS or multivariable modeling. There is potential for missing or unreported data that may not be accessible with chart review. Further trials are needed to in a larger cohort of patients to analyze the optimal sequencing of ADCs in metastatic breast cancer.

## Conclusion

This single-center, retrospective, cohort study highlights the impact on efficacy and cross-resistance that can occur with the use of ADCs sequentially. The pattern of ADC administration achieved different responses in HR +/HER- and TNBC patients. Further trials to understand optimal sequencing among ADCs as well as relevant biomarkers to guide treatment sequencing and gauge response is imperative.

## Supplementary Information

Below is the link to the electronic supplementary material.

Supplementary file1 (DOCX 28 KB)

## Data Availability

No datasets were generated or analysed during the current study.
