# Supplementary material for: Evaluation of efficacy and safety of sequential antibody drug conjugates (ADCs) in human epidermal growth factor 2 (HER2)-negative metastatic breast cancer
Source: Breast Cancer Res Treat. 2025 Sep 11;214(3):329–37. doi: 10.1007/s10549-025-07818-z (PMC12583276; doi:10.1007/s10549-025-07818-z)
Supplement: Supplementary file 1 — Supplementary file1 (DOCX 28 KB) [file 10549_2025_7818_MOESM1_ESM.docx]

**Figure S1. Study Enrollment**

123 patients with HR+/HER2-negative or TNBC breast cancer who received T-DXd and/or SG from April 1, 2021, to December 1, 2023

112 patients were included

11 patients were excluded because they were treated at an outside hospital

59 patients had HR+ MBC

43 patients had TNBC MBC

**Table S1. Previous Chemotherapy**

| **Previous Chemotherapy** | **Total (n=112)** |
| --- | --- |
| Capecitabine | 58 (51.8) |
| Doxil | 13 (11.6) |
| Eribulin | 6 (5.4) |
| Vinorelbine | 8 (7.1) |
| Paclitaxel or Abraxane/Pembrolizumab | 6 (5.4) |
| Carboplatin/Gemcitabine/Pembrolizumab | 13 (11.6) |
| Paclitaxel or Abraxane monotherapy | 33 (29.5) |
| PARPi | 12 (10.7) |
| Pembrolizumab monotherapy | 10 (8.9) |
| Carboplatin/Gemcitabine | 20 (17.9) |
| Paclitaxel/Abraxane/Atezolizumab | 7 (6.3) |
| Carboplatin/Paclitaxel | 7 (6.3) |
| Carboplatin monotherapy | 9 (8.0) |
| Docetaxel monotherapy | 3 (2.7) |
| THP (Docetaxtel/Herceptin/Perjeta) | 3 (2.7) |
| HP (Herceptin/Perjeta) | 4 (3.6) |
| CMF (Cyclophosphamide/Methotrexate/Fluorouracil) | 3 (2.7) |
| Gemcitabine monotherapy | 2 (1.8) |
| TDM-1 | 3 (2.7) |

**Table S2. Previous Endocrine Therapy**

| **Previous Endocrine Therapy** | **Total (n=112)** |
| --- | --- |
| Everolimus/Exemestane | 24 (21.4) |
| Alpelisib/Fulvestrant) | 8 (7.1) |
| CDK4/6i + ET | 50 (44.6) |
| Fulvestrant monotherapy | 12 (10.7) |
| Fulvestrant/Everolimus | 2 (1.8) |
| Anastrozole | 2 (1.8) |
| Tamoxifen | 2 (1.8) |

**Table S3. Repeat Adverse Events in T-DXd Group**

| **Repeat Adverse Events** | **Total (n=48)** |
| --- | --- |
| Repeat adverse event – n (%) | 4 (8.3) |
| Neutropenia – n (%)  Grade 3 | 1 (2.1)  1 (100.0) |
| Nausea – n (%)  Grade 1  Grade 2 | 2 (4.2)  1 (50.0)  1 (50.0) |
| Vomiting – n (%)  Grade 1 | 1 (2.1)  1 (100.0) |

**Table S4. Repeat Adverse Events in SG Group**

| **Repeat Adverse Events – SG Group** | **Total (n=52)** |
| --- | --- |
| Repeat adverse event – n (%) | 7 (13.5) |
| Neutropenia – n (%)  Grade 1  Grade 3 | 3 (5.8)  1 (33.3)  2 (66.7) |
| Nausea – n (%)  Grade 2 | 1 (1.9)  1 (100.0) |
| Diarrhea – n (%)  Grade 1  Grade 2 | 3 (5.8)  1 (33.3)  2 (66.7) |

**Table S5. Chemotherapy Initiated After ADC1**

|  | Chemotherapy following TDxd (N=23) | Chemotherapy following SG (N=16) |
| --- | --- | --- |
| **Chemotherapy – n (%)** |  |  |
| Capecitabine | 1 (4.3) | 2 (12.5) |
| Doxil | 10 (43.5) | 3 (18.8) |
| Eribulin | 4 (17.4) | 6 (37.5) |
| Vinorelbine | 1 (4.3) | -- |
| Carboplatin/Gemcitabine/Pembrolizumab | 1 (4.3) | -- |
| Paclitaxel/Abraxane monotherapy | 2 (8.7) | -- |
| PARPi | 1 (4.3) | 1 (6.3) |
| Carboplatin monotherapy | -- | 2 (12.5) |
| Carboplatin/Gemcitabine | 1 (4.3) | 2 (12.5) |
| Elacestrant | 2 (8.7) | -- |

**Table S6. Chemotherapy Initiated After ADC1 and ADC2**

| **Chemotherapy – n (%)** | Total (n=7) |
| --- | --- |
| Capecitabine | 2 (28.6%) |
| Eribulin | 1 (14.3%) |
| Carboplatin/Gemcitabine | 2 (28.6%) |
| Vinorelbine/Herceptin | 1 (14.3%) |
| Elacestrant | 1 (14.3%) |
